# The relationship between unhealthy lifestyle patterns and depressive symptoms among residents in Beijing, China: A community-based cross-sectional study

**DOI:** 10.3389/fpubh.2023.1055209

**Published:** 2023-04-12

**Authors:** Xiaoyue Zhang, Lanchao Zhang, Yihua Liu, Yuxin Lin, Xiaochen Yang, Litong Gong, Chun Chang

**Affiliations:** ^1^Department of Social Medicine and Health Education, School of Public Health, Peking University Health Science Center, Beijing, Haidian District, China; ^2^Department of Daxing Center for Disease Control and Prevention, Beijing, Daxing District, China

**Keywords:** depressive symptoms, unhealthy lifestyles, China, behavioral combinations, patient health questionnaire

## Abstract

**Introduction:**

Depression is a prevalent mental disorder that has an irreversible impact on people’s health status. Unhealthy lifestyles are modifiable and influence mental health significantly. The purpose of this study was to explore the impact of different unhealthy lifestyles and their patterns on depressive symptoms.

**Methods:**

The data for this study were obtained from the 2017 Community Diagnostic survey in Daxing District, Beijing. It was a cross-sectional study that included 6,252 samples. The Patient Health Questionnaire version 9 was used to measure depressive symptoms, and the self-administered questionaires were used to investigate five unhealthy lifestyles, including sleep deprivation, the inadequate intake of fruits and vegetables, physical inactivity, smoking, and excessive alcohol consumption. Respondents were assigned 1 point for each of their unhealthy lifestyles, and their overall unhealthy lifestyle scores were calculated. The total scores of unhealthy lifestyles ranged from 0 to 5. Descriptive analyses and Firth’s logistic regression model were used to analyze the relationship between unhealthy lifestyle and depression symptoms.

**Results:**

It was found that 12.1% of the participants had depressive symptoms. The respondents whose unhealthy lifestyle scores were 2 (OR1.45, 95%CI:1.01 to 2.12), 3 (OR2.29, 95%CI:1.57 to 3.42), 4 (OR 3.04, 95%CI:1.96 to 4.76), or 5 (OR4.08, 95%CI:2.09 to 7.78) were more likely to experience depressive symptoms in comparison with those whose unhealthy lifestyle scores were 0, and the OR increased with the unhealthy lifestyle scores. When the participants had 3 or more unhealthy lifestyles at the same time, different combination patterns of unhealthy lifestyles showed a different effect on depression. The OR was 3.01 (95%CI:1.45 to 5.95) for the combination of sleep deprivation—insufficient intake of fruit and vegetables—excessive alcohol consumption, and was 2.89 (95%CI:1.52 to 5.25) for the combination of sleep deprivation—insufficient intake of fruit and vegetables—physical inactivity—excessive alcohol consumption.

**Discussion:**

The co-existence of multiple unhealthy behavioral lifestyles are associated with depressive symptoms. Among the five unhealthy lifestyles, sleep deprivation and the inadequate intake of fruits and vegetables may have a greater impact on depression.

## 1. Introduction

The process of modern life is accelerating, people suffer more pressure from life and work, and most people maintain a status of psychological sub-health (1). Depression as a common mental disorder, has been increasing in prevalence and disease burden in recent years (2, 3). According to a global survey conducted in 2020, severe depressive disorder has increased by 28% (4). Data from a nationwide survey found that the lifetime prevalence of depressive disorder was 6.8% (5).

Depressive symptoms are specific manifestations of depression, including unhappiness, loss of pleasure in everything, lack of energy, and physical and cognitive impairments (6). A study identified that 31.0% of Chinese adults showed depressive symptoms, with the prevalence rate exceeding that of depression (7). In addition, due to factors such as life expectancy and social status, women faced more risk of depressive symptoms than men (8). Unlike the disorder, depressive symptoms may only last a short time or occur in response to a specific event, and are easier to manage and change than the illness (9). The continued aggravation of depressive symptoms will eventually lead to the occurrence of depression, with serious consequences, such as loss of hope in life, and even suicide (6). However, depressive symptoms are often overlooked due to cognitive deficiencies and disease discrimination. Furthermore, they are, in fact, important for the early identification and treatment of the disorder (10).

Among the complicated causes of depression, lifestyle factors are closely associated with depressive symptoms (11, 12). The impact of lifestyles on depressive symptoms is multidimensional and not limited to prevention and treatment. A growing number of studies have shown that certain behaviors, including smoking, alcohol consumption, exercise, diet, and sleep, are important influences on the prevalence of depression and that maintaining two to three healthy behaviors can improve mental health (13, 14). For example, studies point to the fact that a Mediterranean diet, which includes eating sufficient vegetables and fruits, fish, and grains, lowers the risk of depression; while insufficient sleep, alcohol abuse, and depression severity are positively associated (15, 16). Michas et al. (17) indicated that regular physical activity can effectively prevent and reduce depressive symptoms. In addition, there is a bidirectional relationship between depression and unhealthy lifestyles, and the occurrence of depression may also exacerbate unhealthy behavioral lifestyles. For example, depression may lead to the sleep disorders, and may also lead individuals to develop barriers to smoking or alcohol cessation (18, 19).

At the same time, unhealthy lifestyles often coincide, modulate, and interact with each other and behavioral superimposition increases the risk of depression (20). A systematic review of behavioral lifestyle interventions for depressed patients showed that sustained interventions for smoking, alcohol consumption, physical activity, sleep, and substance abuse promoted behavioral changes and could improve depressive symptoms (21). Current studies have demonstrated the effect of individual behaviors on depressive symptoms. Lifestyles are modifiable and assessing the effects of different behavioral combinations on depression is essential for understanding their relationship and developing more targeted interventions (18, 22). The purpose of this study was to explore the relationship between depressive symptoms and unhealthy lifestyles. Furthermore, we also assessed the extent to which different behavioral patterns are associated with depressive symptoms. We formed two hypotheses:

a. The number of unhealthy lifestyles should be significantly related to developing depressive symptoms.
b. Different patterns of unhealthy lifestyle combinations associated with participants’ mental health status.

## 2. Method

### 2.1. Data sources

The Beijing Daxing District Community Diagnostic (BDDCD) survey provided the data for this study. BDDCD is a community-based cross-sectional study that aims to investigate the major chronic diseases and risk factors in the general population. The survey, which was designed by Peking University and implemented by the Beijing Daxing District Centers for Disease Control and Prevention, was conducted in Beijing, China, in 2017. The informed consent forms were signed by each participant, and the study was approved by the ethics committee at Peking University. A combination of PPS sampling and quota sampling was used for the BDDCD survey, and a total of 7,019 participants were involved. The inclusion criteria for the study subjects were mainly permanent residents aged 18 years and above in Daxing District; the exclusion criteria were participants with intellectual disability, Alzheimer’s disease, mentally ill patients in the disease phase, and others who could not cooperate. For this study, we ultimately included 6,252 participants (age ≥ 18) after excluding those with missing answers to the main research behavior questions. The structured questionnaire was designed by Peking University, and the main variables included sociodemographic characteristics, lifestyle factors, and mental health status.

### 2.2. Measures

#### 2.2.1. Mental health status

Depressive symptoms were measured using the nine-item Patient Health Questionnaire (PHQ-9), ranging from 0 to 27. The PHQ-9 for the Chinese version has good internal consistency, reliability and validity, with a Cronbach’s 0.86 (23). Various ratings describe the severity of depression symptoms, with 0–4 indicating no symptoms, 5–9 indicating mild symptoms, 10–14 indicating moderate symptoms, and 15–27 indicating severe symptoms (24). For this study, we focused on depressive symptoms rather than the disease, and considering that above 5 is the criterion for determining the presence of depressive symptoms, we set 5 as the cut-off score in this study (24). We separated the participants into two groups, with those scoring 0–4 as having no depression symptoms and those scoring 5 and above as having more than mild depressive symptoms.

#### 2.2.2. Unhealthy lifestyle scores

Based on the application of behavioral scores to evaluate health problems in previous studies (25, 26), we created the unhealthy lifestyle score (ULS) with five elements: sleep deprivation (SD), inadequate intake of fruits and vegetables (IFV), physical inactivity (PIA), smoking (SM), and excessive alcohol consumption (EAC) to determine the magnitude of the combined effect of unhealthy lifestyles on depressive symptoms. A systematic questionnaire was used to assess all behaviors, and each unhealthy lifestyles was assigned a score of 0 or 1. The behavioral score was calculated by adding all the individual behaviors, ranging from 0 to 5. Higher scores meant more unhealthy lifestyles for the individual.

As a measure of smokers, we asked, “Have you ever smoked?” The response options were A: smoked but now have quit, B: still smoking C: never smoked. Nonsmokers and those who have stopped smoking were assigned a score of 0 and 1 was assigned for smokers. The health risks of excessive alcohol consumption are wieldy confirmed (27), and identified drinking behavior by asking, “How often have you been drunk in the past 12 months?” with 5 answers: A ≥ 5 days per week, B ≥ 1–4 days per week, C ≥ 1–3 days per month, D < 1 day per week, and E never. Considering the health effects of different amounts of alcohol consumption, those who answered never, scored 0, and other answers, received 1. The physical activity levels were assessed by the International Physical Activity Questionnaire. According to the WHO’s “Guidelines for Exercise and Sedentary Behavior 2020” (28), adults aged 18 to 64 should do at least 150 min of moderate-intensity exercise per week, 75 min of high-intensity exercise per week, or an equivalent combination of both moderate and high-intensity exercise. And for those who aged ≥65 should get at least 150–300 min of moderate-intensity or at least 75–150 min of high-intensity exercise per week. Accordingly, we divided the participants into two groups based on WHO recommendations for exercise: those who do not meet the standards of physical activity (assigned 1), and those who do (assigned 0). The frequent food questionnaire (FFQ) was utilized to examine the participants’ fruit and vegetable consumption. According to the “Dietary Guidelines for Chinese Residents” (29), adults should consume at least 200 g of fruits and 300 g of vegetables daily. We therefore separated the participants into two groups: those who consume insufficient/no fruits or vegetables (assigned 1) and those who consume sufficient fruits and vegetables (assigned 0). Lack of sleep was defined as less than 6 h per day for adults, according to the National Sleep Foundation’s recommendations (30). Therefore, we divided participants and assigned 1 to the lack of sleep group (sleep duration<6 h) and 0 to the group with sufficient sleep (sleep duration≥6 h).

### 2.3. Covariates

The sociodemographic characteristics, including age (> 30, 30–40, 41–50, 51–60, and ≥ 60), gender (male, female), ethnicity (han ethnic, other), educational attainment (middle school or lower, high school, college and above), marital status (single, married, other), occupational status (yes, other), income level (0–1,409, 1,410–2,999, 3,000–3,999, 4,000–4,999, and ≥ 5,000), and chronic disease were asked about. Moreover, chronic disease was classified into two categories: chronic disease and no chronic disease. The calculation of BMI required the height and weight of the participants, and this data was collected by trained investigators using standard methods of physical testing.

### 2.4. Statistical analysis

The study used univariate and multivariate analyses for each parameter. Categorical variables were reported as frequencies and percentages in univariate analysis, and statistically significant differences were tested using the χ2 test or Fisher’s exact test, with *p* values less than 0.05 considered statistically significant. Firth’s logistic regression is a better method for assessing binary outcomes in small samples and variable separability, and decreases bias in maximum likelihood coefficient estimation. In this study, as depressive symptoms were comparatively rare in the sample, Firth’s logistic regression was used to reduce the statistical bias associated with the separation of outcome variables. Covariates had some missing data, and the missing data were filled using multiple imputations to create 20 imputation datasets under joint multivariate normal imputation.

The univariate analyses and missing data imputed were conducted in Stata version 16.0, and Firth’s logistic regression model was analyzed in R 4.1.2 (logistf package).

## 3. Results

### 3.1. Population characteristics

Table 1 shows the sociodemographic characteristics of the participants in the BDDCD survey. A total of 6,252 participants were involved in this study, and 12.11% have depressive symptoms, 87.9% scored 0–4, 9.72% scored 5–10, and 2.40% scored ≥10. People with symptoms of depression are more likely to be age ≤ 30(56.27%), female (52.68%), bachelor degree or above (49.87%), married (61.34%).

**Table 1:** Sociodemographic of participants in the BDDCD survey according to depressive symptoms (*n* = 6,252).

Variables	Depressive Symptoms [*n*(%)]		Total (*n*)	*P*
	Yes	No		
**Age**				<0.001
≤30	426 (56.27)	1992 (36.25)	2,418	
31–40	137 (18.1)	1,002 (18.23)	1,139	
41–50	81 (10.7)	1,126 (20.49)	1,207	
51–60	54 (7.13)	741 (13.48)	795	
≥60	59 (7.79)	634 (11.54)	693	
**Gender**				0.001
Male	353 (47.32)	2,914 (53.92)	3,267	
Female	393 (52.68)	2,490 (46.08)	2,883	
**Ethnicity**				0.172
Han	713 (94.19)	5,236 (95.32)	5,949	
Other	44 (5.81)	257 (4.68)	301	
**Education attainment**				<0.001
Middle school or lower	195 (25.86)	1956 (35.79)	2,151	
High school	183 (24.27)	1,452 (26.57)	1,635	
Bachelor and above	376 (49.87)	2057 (37.64)	2,433	
**Marriage**				<0.001
Unmarried	261 (35.66)	1,007 (19.31)	1,268	
Married	449 (61.34)	4,047 (77.59)	4,496	
Other	22 (3.01)	162 (3.11)	184	
**Occupation status**				0.968
Yes	563 (75.17)	4,092 (75.23)	4,655	
Other	186 (24.83)	1,347 (24.77)	1,533	
**Income level**				0.069
0–1,409	108 (14.32)	920 (16.81)	1,028	
1,410–2,999	150 (19.89)	1,228 (22.44)	1,378	
3,000–3,999	198 (26.26)	1,310 (23.94)	1,508	
4,000–4,999	97 (12.86)	727 (13.28)	824	
≥5,000	201 (26.66)	1,288 (23.53)	1,489	
**Chronic disease**				0.939
Yes	297 (39.97)	2,165 (39.83)	2,462	
No	446 (60.03)	3,271 (60.17)	3,717	
**Total**	757 (12.11)	5,495 (87.89)	6,252	

Values are numbers (percentages) unless stated otherwise. Several of the socio-demographic factors had missing values, and the missing values were filled by multiple imputations.

### 3.2. Unhealthy lifestyle score and unhealthy lifestyles

**Figure1:**
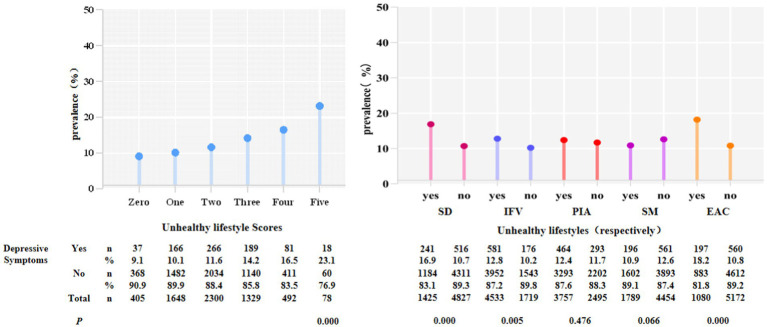
Participants in the BDCCD with depressive symptoms by ULS and unhealthy lifestyles, respectively (*p* < 0.05). SD, sleep deprivation; IFV, inadequate intake of fruits and vegetables; PIA, physical inactivity; SM, smoking; EAC, excessive alcohol consumption.

Figure 1 shows the prevalence of depressive symptoms among different ULS and unhealthy lifestyles. In the survey, the prevalence of depressive symptoms varied with increases in ULS, with the lowest percentage of depressive symptoms at 9.1% (*n* = 37) for 0, and the highest percentage of depressive symptoms at 23.1% (*n* = 18) for 5. The difference between the ULS were statistically significant (*p* < 0.05). The corresponding figures for the other groups were 10.1% (*n* = 166) for 1, 11.6% (*n* = 266) for 2, 14.2% (*n* = 189) for 3, and 16.5% (*n* = 81) for 4 and 23.1% (*n* =18) for 5, respectively.

For individual unhealthy lifestyles, participants with sleep deprivation (SD) [*n* = 241 (16.9%)], inadequate intake of fruits and vegetables (IFV) [*n* = 581 (12.8%)], physical inactivity (PIA) [*n* = 464 (12.4%)], and excessive alcohol consumption (EAC) [*n* = 197 (18.2%)] were more likely to have depressive symptoms than those who did not [*n* = 516 (10.7%), *n* = 176 (10.2%), *n* = 293 (11.7%), *n* = 560 (10.8%)]. Regarding smoking (SM), non-smokers [*n* = 561 (12.6%)] were more likely to have depressive symptoms. The differences between behaviors, with the exception of physical inactivity and smoking, were statistically significant (*p* < 0.05).

### 3.3. Association between unhealthy lifestyle score and depressive symptoms

**Figure 2:**
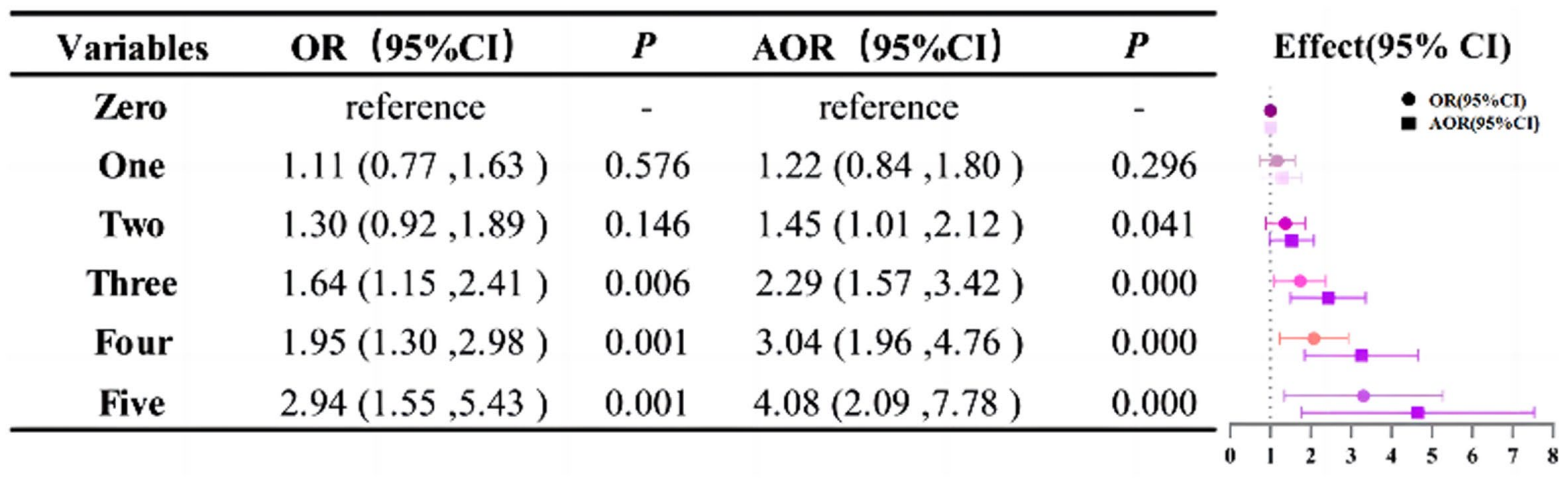
The relationship between depressive symptoms and ULS. The Firth’s logistic models were adjusted for age, gender, ethnicity, educational attainment, marriage, occupational status, income level, chronic disease, and BMI.

The results of Firth’s logistic regression model revealed that ULS were associations for depressive symptoms before and after adjustment. Compared to 0 points, after controlling for age, gender, ethnicity, educational attainment, marriage, occupational status, income level, chronic disease, and BMI, 2 points (AOR 1.45, 95% CI:1.01 to 2.12), 3 points (AOR 2.29, 95% CI:1.57 to 3.42), 4 points (AOR 3.04, 95% CI:1.96 to 4.76), and 5 points (AOR 4.08, 95% CI:2.09 to 7.78) were more likely to have depressive symptoms (*p* < 0.05). There was no significant difference in 1 point (AOR 1.22, 95% CI:0.84 to 1.80; Figure 2).

### 3.4. Association between individual unhealthy lifestyles and depressive symptoms

**Figure 3:**
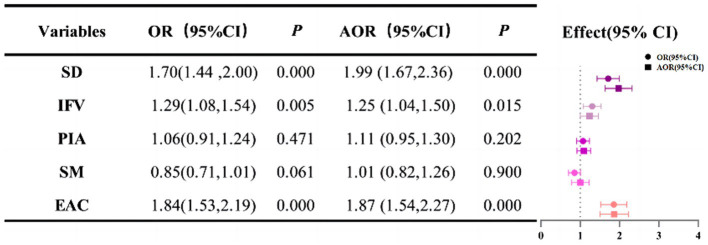
The relationship between depressive symptoms and unhealthy lifestyles, respectively. SD, sleep deprivation; IFV, inadequate intake of fruits and vegetables; PIA, physical inactivity; SM, smoking; EAC, excessive alcohol consumption The Firth’s logistic models were adjusted for age, gender, ethnicity, educational attainment, marriage, occupational status, income level, chronic disease and BMI.

After adjusting the covariates, unhealthy lifestyle including sleep deprivation (AOR 1.99, 95%CI:1.67 to 2.36), inadequate intake of fruits and vegetables (AOR 1.25, 95%CI:1.04 to 1.50) and excessive alcohol consumption (AOR 1.87, 95%CI:1.54 to 2.27), were all risk factors for depressive symptoms. However, no statistical differences were observed between physical inactivity and smoking (Figure 3).

### 3.5. Association between unhealthy lifestyle patterns and depressive symptoms

**Figure 4:**
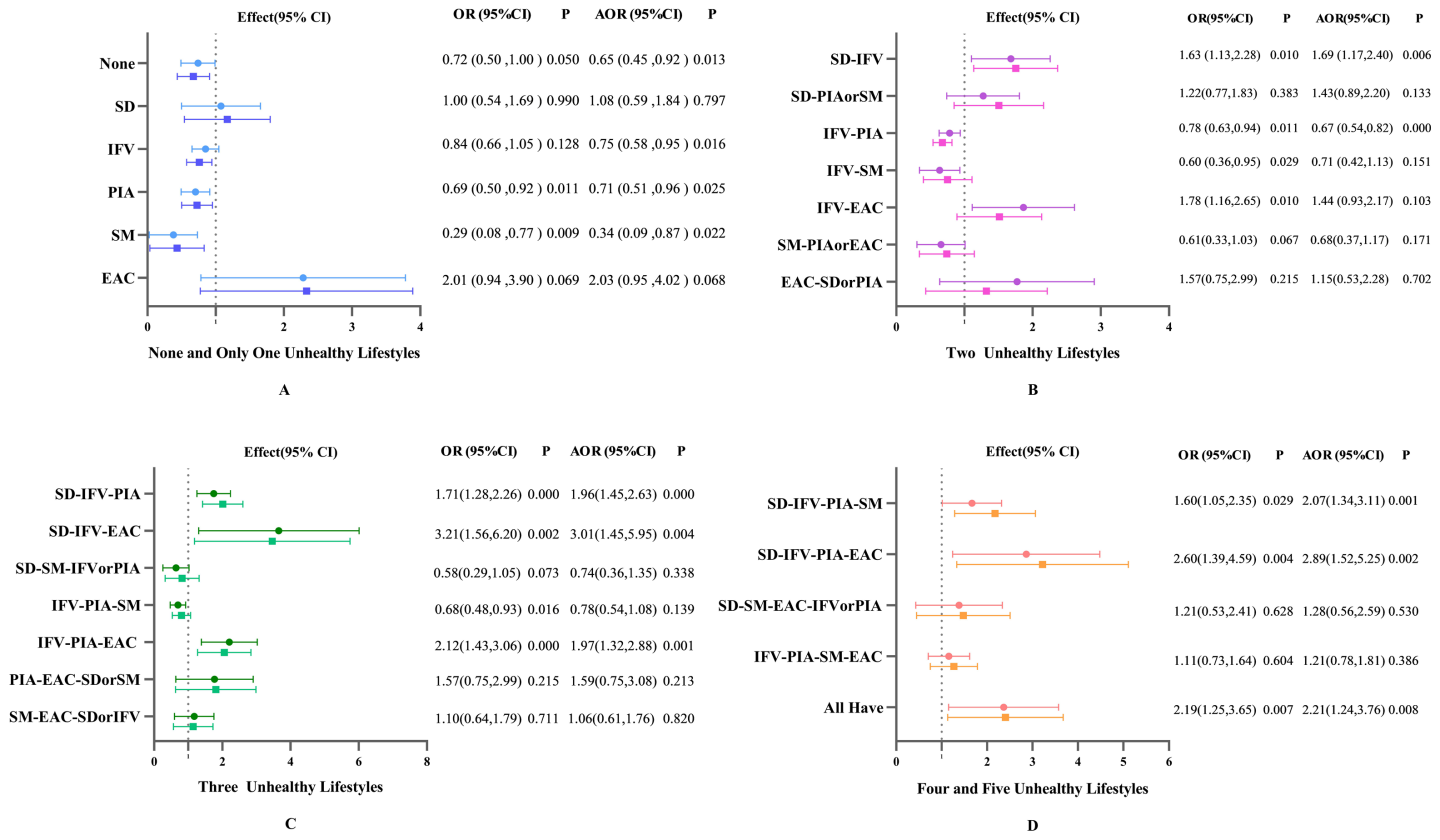
Odds ratios (OR) and 95% confidence intervals of unhealthy lifestyle patterns in relation to depressive symptoms. **(A)** shows the patterns of having no or only one unhealthy lifestyle, **B** shows the patterns of having two of unhealthy lifestyles, **C** shows the patterns of having three unhealthy lifestyles, and **D** shows the the patterns of having more than four unhealthy lifestyles. SD, sleep deprivation; IFV, inadequate intake of fruits and vegetables; PIA, physical inactivity; SM, smoking; EAC, excessive alcohol consumption. The Firth’s logistic models were adjusted for age, gender, ethnicity, educational attainment, marriage, occupational status, income level, chronic disease and BMI. Some patterns are too small in number, so they are combined. In patterns with two unhealthy lifestyles, SD-PIA and SD-SM are combined and denoted as SD-PIA or SM; SM-PIA and SM-EAC are denoted as SM-PIA or EAC; EAC-SD and EAC-PIA are denoted as EAC-SD or PIA. In patterns with three unhealthy lifestyles, SD-SM-IFV and SD-SM-PIA are combined and denoted as SD-SM-IFV or PIA; PIA-EAC-SD and PIA-EAC-SM are denoted as PIA-EAC-SD or SM; SM-EAC-SD and SM-EAC-IFV are denoted as SM-EAC-SD or IFV. In patterns with four unhealthy lifestyles, SD-SM-EAC-IFV and SD-SM-EAC-PIA are combined and denoted as SD-SM-EAC-IFV or PIA.

In each dimension, Figure 4 depicts the relationship between unhealthy lifestyle patterns and depressive symptoms. After controlling for covariates, no unhealthy lifestyle (AOR 0.65, 95%CI:0.45 to 0.92), only inadequate intake of fruits and vegetables (IFV; AOR 0.75, 95%CI:0.58 to 0.95), only physical inactivity (PIA; AOR 0.71, 95%CI:0.51 to 0.96), and only smoking (SM; AOR 0.34, 95%CI:0.09 to 0.87) were shown to be negatively associated with depressive symptoms. SD-IFV (AOR 1.69, 95%CI:1.17 to 2.40) were the risk factors for depressive symptoms in the pattern with two behavioral risk variables, and IFV-PIA (AOR 0.67, 95%CI:0.54 to 0.82) were inversely related to depressive symptoms. Others were not statistically significant.

For the patterns with three and four risk behaviors, SD-IFV-PIA (AOR 1.96, 95%CI:1.45 to 2.63), SD-IFV-EAC (AOR 3.01 95%CI:1.45 to 5.95), IFV-PIA-EAC (AOR 1.97, 95%CI:1.32 to 2.88), SD-IFV-PIA-SM (AOR 2.07, 95%CI:1.34 to 3.11), SD-IFV-PIA-EAC (AOR 2.89, 95%CI:1.52 to 5.25) were risk factors for depressive symptoms. Furthermore, having all unhealthy lifestyles (AOR 2.21, 95%CI:1.24 to 3.76) was also a risk factor (*p* < 0.05).

## 4. Discussion

Constructing unhealthy lifestyle scores to reveal the impact of different behavioral patterns on depressive symptoms allowed for the relationship between them to be understood from a comprehensive perspective (26). The main findings suggested that a total of 12.11% of the participants in this sample had depressive symptoms and that sleep deprivation, inadequate intake of fruits and vegetables, and excess alcohol consumption were all risk factors for depressive symptoms, but no association was found between physical inactivity and smoking and depressive symptoms.

Furthermore, the number of unhealthy lifestyle behaviors was positively associated with the risk of depressive symptoms (31). When the number of individual unhealthy behaviors reaches three or more in behavioral pattern studies, a negative impact can be observed on the individual’s depressive symptoms. Werneck et al. (32) demonstrated that the risk of developing depressive symptoms was nearly two times higher when individuals had more than three poor lifestyles than those who did not. Fukunaga and Adjibade et al. (31, 33) confirmed the effect of behavior on depression by constructing the Healthy Lifestyle Index (HLI), and found that every 1-point increase in HLI can reduce 10% of the risk of depressive symptoms. However, BMI was included in the HLI of their studies which was not included in our study. Behaviors often do not exist independently, they modulate each other to interact, and when multiple unhealthy lifestyles coexist, the additive effect that results has a greater impact on the individual (34).

In addition, by analyzing the relationship between different combinations of behavioral risk factors and depressive symptoms, we found that sleep deprivation or vegetable and fruit deficiency had a greater effect than other behavioral combinations. The present study found that individuals with insufficient sleep time are nearly twice as likely to experience depressive symptoms as those with adequate sleep, consistent with previous research findings (35). Adequate and high-quality sleep has been found in studies to minimize the incidence of depression and ameliorate depressed symptoms (36, 37). Dong et al. (38) discovered a U-shaped relationship between sleep and depressive events, with sleep duration and depression being negatively correlated when it was less than 8 h and associated with an increased risk of depression when it was greater than 8 h. Another longitudinal study found that after controlling for confounders, a higher incidence of depressive symptoms and recurrence were present in the population which sleep less than 6 h (39). Relevant studies have pointed out a bidirectional association between sleep and depression, depression may exist before sleep deprivation, and depressive symptoms may also lead to insomnia (40). The mechanism between sleep and depression is complex. The two share some common neurological and physio-pathological mechanisms, and sleep disturbance promotes an increase in inflammation, which leads to an increased risk of depression (39, 41).

We also found that diet is an essential factor for the mental health status of individuals. The Mediterranean diet effectively prevents depression, with high intakes of vegetables, fruits, and fish showing a substantial protective effect (42, 43). A systematic review discovered that vegetables and fruits are rich in vitamin C, vitamin B, fiber, and other nutrients and that the intake of these substances not only ensures the body’s required nutrients but also has anti-inflammatory and antioxidant effects that influence the outcome of the body’s metabolic functions and have an important impact on a person’s mental health (44). One study showed that increasing a fruit or vegetable diet by 100 g lowers the prevenance of depression by 5% (45). Furthermore, various studies have confirmed that folic acid impacts the nervous system and that the intake of substances such as omega-3 fatty acids has an anti-inflammatory effect, influencing the synthesis of neurotransmitters and thus effectively reducing the risk of depression (46). Additionally, we found a link between excessive alcohol consumption and depressive symptoms. Tsai et al. (12) showed that people who drink excessively have a 1.8 times higher risk of depressive symptoms than the general population, but moderate alcohol consumption may not be associated. Binge drinking can damage the brain and may exacerbate stressors such as unemployment, debt, failed marriages, and the death of loved ones (47, 48). Furthermore, it is easy to panic or become overexcited after drinking, and emotional instability can exacerbate depressive symptoms (49).

Liu et al. (19) provided evidence of dose–response relationships between the cigarette with depressive symptoms and those who light and mild smoking were more likely to have depressive symptoms compared to non-smokers. In contrast to previous research, the current study found no correlation between smoking and depressive symptoms. The higher rate of depressive symptoms among nonsmokers than smokers in this study may have contributed to such results. Xu et al. (50) found no significant differences in depression scores between the groups of smokers and nonsmokers after adjusting for confounding factors, which may be due to the fact that the relationship between smoking and depression is likely to be bidirectional, with smoking possibly serving as a form of stress and symptom relief over a period of time. However, a cohort study carried out in France found that smoking raises inflammatory markers in the body and is a risk factor for depression (33). The association between physical activity and depressive symptoms was also not identified in this study, which might be because we focused on symptoms rather than the diagnosed disease. In addition, in nonclinical patients, the effects of physical activity were not as strong as in clinical patients, and physical activity differed from exercise, with most people having adequate physical activity in daily living but lacking exercise (33, 40).

This study had some limitations. First, because this was a cross-sectional study, it was unable to reveal the causal relationship between behavioral factors and depressive symptoms; second, this study only used one area of Beijing as a sample, which means that it reflects only a specific situation in that area; third, because of the small sample size, the numbers of each behavioral pattern were limited, and while a reasonable statistical approach was used, statistical bias could not be avoided, and more sample sizes are required for future research; fourth, part of the questionnaire was in the form of self-reporting, which makes it difficult to avoid recall bias; fifth, the effect of different behaviors on depressive symptoms is variable, and we did not consider the power of individual behavior scores when constructing the behavioral risk factor indicator; finally, depressive symptoms are different from depression, and this study mainly focused on the relationship between symptoms and behavior. Therefore, further research is required in the future to explain the association between depression and behaviors.

## 5. Conclusion

By constructing the ULS, we found that unhealthy lifestyles have a significant impact on the prevalence of depressive symptoms, and when multiple behaviors coexist, they often interact to exacerbate the depressive symptoms, especially when the number of unhealthy lifestyles is three or more. Furthermore, we found that sleep, diet and alcohol consumption have an impact on the depressive symptoms. In the future, individualized behavioral change goals should be formulated based on individual circumstances, focusing on the impact of key behaviors on individuals. Greater emphasis on specific behaviors and targeted interventions to prevent depression and promote mental health is required.

## Data availability statement

The raw data supporting the conclusions of this article will be made available by the authors, without undue reservation.

## Ethics statement

The studies involving human participants were reviewed and approved by the Ethics Commission of Peking University Health Science Centre. The patients/participants provided their written informed consent to participate in this study.

## Author contributions

XZ finished the designed and written of the manuscript. LZ, YHL, YXL, XY, and LG conducted the data analysis. CC managed the study. All authors have read and approved the final manuscript.

## Funding

This study was supported by Peking University.

## Conflict of interest

The authors declare that the research was conducted in the absence of any commercial or financial relationships that could be construed as a potential conflict of interest.

The reviewer YZ declared a shared affiliation with the authors XZ, LZ, YHL, YXL, XY, LG, and CC to the handling editor at the time of review.

## Publisher’s note

All claims expressed in this article are solely those of the authors and do not necessarily represent those of their affiliated organizations, or those of the publisher, the editors and the reviewers. Any product that may be evaluated in this article, or claim that may be made by its manufacturer, is not guaranteed or endorsed by the publisher.
